# A high-performance computational workflow to accelerate GATK SNP detection across a 25-genome dataset

**DOI:** 10.1186/s12915-024-01820-5

**Published:** 2024-01-25

**Authors:** Yong Zhou, Nagarajan Kathiresan, Zhichao Yu, Luis F. Rivera, Yujian Yang, Manjula Thimma, Keerthana Manickam, Dmytro Chebotarov, Ramil Mauleon, Kapeel Chougule, Sharon Wei, Tingting Gao, Carl D. Green, Andrea Zuccolo, Weibo Xie, Doreen Ware, Jianwei Zhang, Kenneth L. McNally, Rod A. Wing

**Affiliations:** 1 Center for Desert Agriculture (CDA), Biological and Environmental Sciences & Engineering Division (BESE), King Abdullah University of Science and Technology (KAUST), Thuwal, 23955-6900 Saudi Arabia; 2 Arizona Genomics Institute (AGI), School of Plant Sciences, University of Arizona, Tucson, AZ 85721 USA; 3 KAUST Supercomputing Laboratory (KSL), King Abdullah University of Science and Technology (KAUST), Thuwal, 23955-6900 Saudi Arabia; 4 National Key Laboratory of Crop Genetic Improvement, Hubei Hongshan Laboratory, Huazhong Agricultural University, Wuhan, 430070 China; 5 International Rice Research Institute (IRRI), Los Baños, Laguna, 4031 Philippines; 6 Cold Spring Harbor Laboratory, Cold Spring Harbor, NY 11724 USA; 7 Information Technology Department, King Abdullah University of Science and Technology (KAUST), Thuwal, 23955-6900 Saudi Arabia; 8 Crop Science Research Center (CSRC), Scuola Superiore Sant’Anna, Pisa, 56127 Italy; 9 USDA ARS NEA Plant, Soil & Nutrition Laboratory Research Unit, Ithaca, NY 14853 USA

**Keywords:** High-performance computing (HPC), Genome Analysis Toolkit (GATK), Single-nucleotide polymorphisms (SNPs), Rice, Sorghum, Maize, Soybean

## Abstract

**Background:**

Single-nucleotide polymorphisms (SNPs) are the most widely used form of molecular genetic variation studies. As reference genomes and resequencing data sets expand exponentially, tools must be in place to call SNPs at a similar pace. The genome analysis toolkit (GATK) is one of the most widely used SNP calling software tools publicly available, but unfortunately, high-performance computing versions of this tool have yet to become widely available and affordable.

**Results:**

Here we report an open-source high-performance computing genome variant calling workflow (HPC-GVCW) for GATK that can run on multiple computing platforms from supercomputers to desktop machines. We benchmarked HPC-GVCW on multiple crop species for performance and accuracy with comparable results with previously published reports (using GATK alone). Finally, we used HPC-GVCW in production mode to call SNPs on a “subpopulation aware” 16-genome rice reference panel with ~ 3000 resequenced rice accessions. The entire process took ~ 16 weeks and resulted in the identification of an average of 27.3 M SNPs/genome and the discovery of ~ 2.3 million novel SNPs that were not present in the flagship reference genome for rice (i.e., IRGSP RefSeq).

**Conclusions:**

This study developed an open-source pipeline (HPC-GVCW) to run GATK on HPC platforms, which significantly improved the speed at which SNPs can be called. The workflow is widely applicable as demonstrated successfully for four major crop species with genomes ranging in size from 400 Mb to 2.4 Gb. Using HPC-GVCW in production mode to call SNPs on a 25 multi-crop-reference genome data set produced over 1.1 billion SNPs that were publicly released for functional and breeding studies. For rice, many novel SNPs were identified and were found to reside within genes and open chromatin regions that are predicted to have functional consequences. Combined, our results demonstrate the usefulness of combining a high-performance SNP calling architecture solution with a subpopulation-aware reference genome panel for rapid SNP discovery and public deployment.

**Supplementary Information:**

The online version contains supplementary material available at 10.1186/s12915-024-01820-5.

## Background

Single-nucleotide polymorphisms (SNPs) are one of the most common types of genetic variation (e.g., SNPs, insertions, deletions, copy number variations, and inversions) used to study genetic diversity among living organisms [1, 2], and are routinely detected by mapping resequencing data to reference genomes using various software tools [3–5]. In major crops, SNPs are routinely discovered using genome resequencing or array-based hybridization methods on thousands of accessions as documented for rice [6, 7], maize [8], soybean [9], and sorghum [10]. In order for such data to be used more widely for trait discovery, genomic selection, and functional genomics applications, numerous databases have been developed for crop plants such as, e.g., SNP-Seek [11], ViceVarMap [12], MaizeSNPDB [13], and RiceNavi [14]. Unfortunately, as crop communities continue to improve their flagship genome assemblies, as well as produce multiple new assemblies that take into account population structure [15–17], and other factors, it is becoming more onerous for such databases to keep pace with the onslaught of new data coming online.

The Genome Analysis Toolkit (GATK) [18, 19], one of the most popular software tools developed for SNP identification, has been widely used for SNP detection for many species [9, 20], and was recently modified to identify copy number variants (CNVs) in human [21]. Although vast amounts of resequencing data have been processed using GATK [6, 9, 20–22], the processing speed of the publicly available open-source version(s) can be very time-consuming when very large resequencing data sets are involved. For example, it took our consortia almost 6 months to call SNPs with GATK using ~ 3000 resequenced rice accessions mapped to a single reference genome. Although several commercially and publicly available workflows (e.g., Sentieon [23], Clara Parabricks [24], Falcon [25], DRAGEN-GATK [26]) are now available that accelerate GATK processing times, all require special and expensive hardware (e.g., graphics processing units, GPUs; field-programmable gate arrays, FPGAs) and are normally not suitable for processing large population datasets.

To address the need to detect genetic variation on the almost daily release of high-quality genome assemblies we have identified three challenges that must be solved to meet the demand for speed and efficiency of SNP detection. First, the exponential increase in sequencing and resequencing data requires intelligent data management solutions [23–25] and compressed data formats to reduce storage [26, 27]; second, data analysis needs flexible workflows and monitoring tools for high-throughput detection and debugging [28]; and third, modern high-performance computing (HPC) architectures are needed to complete jobs efficiently [29, 30].

To address these challenges, we designed a flexible workflow and employed high-performance computing (HPC) architectures to develop an open-source genome variant calling workflow for GATK (i.e., HPC-GVCW). The workflow was divided into four phases that include a data parallelization algorithm — “Genome Index splitter” (GIS) [31] — that divides genomes into megabase (MB) size chunks for parallel GATK processing and file merging. By dividing genomes into 45 Mb, 10 Mb, and 5 Mb chunks, we found that the smallest chunk size tested gave the optimal performance. Using HPC-GVCW with a chunk size of 5 Mb enabled us to call SNPs from ~ 3000 resequenced rice accessions (with 17 × genome coverage) on a single rice genome (GS ~ 400 Mb) in 120 h, which is almost ~ 36 times faster than previously reported (~ 6 months).

To demonstrate utility, we ran HPC-GVCW on a 25 crop genomes dataset using publicly available resequencing data sets and the most up-to-date (near) gap-free reference genome releases available and called an average of 27.3 M, 32.6 M, 169.9 M, and 16.2 M SNPs for rice (GS ~ 400 Mb), sorghum (GS ~ 700 Mb), maize (GS ~ 2400 Mb), and soybean (GS ~ 1100 Mb), respectively.

To demonstrate the novelty of the genetic variation discovered, our analysis of SNP datasets from a 16-genome “subpopulation-aware” rice reference panel revealed a total of ~ 2.3 M (8.8%) novel SNPs in total that have yet to be publicly released based. Analysis of these novel SNPs identified 1.3 M SNPs in genes, 20% (i.e., 248,403) of which are predicted to have impacts on gene function. Analysis of open chromatin regions (OCRs) of one accession (i.e., Zhenshan 97) revealed the presence of 7441 novel SNP that may have effects on gene regulation. Finally, in a test case to evaluate the allele status of known agriculturally important genes, we identified 180 accessions that contain the submergence tolerant allele in the *Sub1A* gene that could be integrated into accelerated breeding programs.

## Results

### HPC-GVCW development

**Fig. 1:**
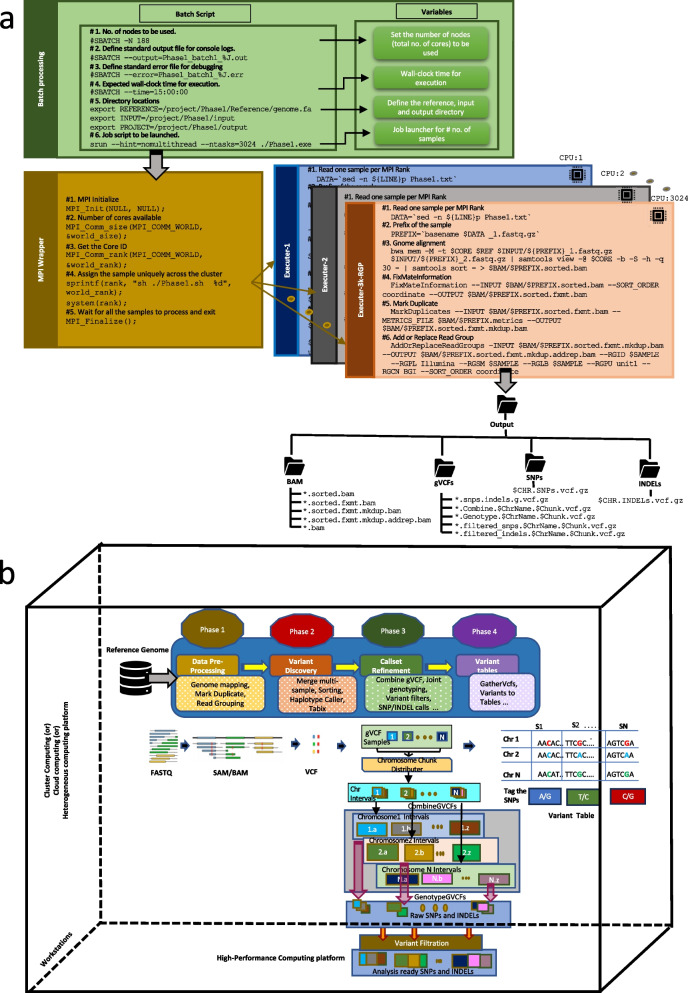
Automated and flexible genome variant calling workflow (GVCW) design for **a** HPC systems and **b** diversified system architectures

HPC-GVCW was designed into four phases: (1) mapping, (2) variant calling, (3) call set refinement and consolidation, and (4) variant merging (Fig. 1). Briefly, Phase 1 was designed to map clean resequencing reads to a reference genome. Phase 2 was designed to call variants using GATK for each sample. Phase 3 was designed to merge all variants per sample into a non-redundant joint genotype file by genome-wide intervals (also called “chunks”). Phase 4 was designed to generate a genome-wide joint genotype by assembling all variant intervals (detailed in Additional file 1: Automated Genome Variant Calling Workflow Design [32–35]; Additional file 2: Fig. S1; Additional file 3: Table S1). The GVCW workflow was designed to run on high-performance computers (Fig. 1a); however, it can also be employed on alternative computational platforms, including hybrid clusters and high-end workstations (Fig. 1b). Of note, each of the four phases is independent of one another, flexible, and scalable across multiple nodes and platforms (Additional file 1: Workflow flexibility).

With this workflow, the most challenging component to address was the merging of large sample sets (e.g., 3000 rice accessions) into a joint file using GATK with a single node, i.e., Phase 3. To address this challenge, we modified the “genome intervals joint genotype” module supported by GATK (“CombineGVCFs” and “GenotypeGVCFs,” detailed in Additional file 1: Automated Genome Variant Calling Workflow Design) by adding an algorithm called “Genome Index Splitter” (GIS) [31] that can optimize the size and number of genomics intervals utilized. The GIS algorithm creates a “chromosome split table” (CST) to index disjoint variant intervals, which can be fine-tuned based on genome size and available “central processing units” (CPUs) (Additional file 2: Fig. S1c–d). Optimal chunks are calculated based on three steps: (1) locate the largest chromosome length in a given reference genome; (2) calculate the fairness of a divisible integer for a given maximum number of cores; and (3) whole genome reference sequences are divided by the optimal integer number, as illustrated in Additional file 2: Fig. S1e.

For example, the CST with the entries as follows: < chromosome name (ChrName), chunk number (Chunk_no), chromosome starting position (Start), chromosome end position (End) > (ChrName, Chunk_no, Start, End).

- Chr01 1 1 2277417
- Chr01 2 2277418 4554834
- Chr01 3 4554835 6832251
- Chr01 4 6832252 9109668
- Chr01 5 9109669 11387085
- Chr02 1 1 2277417
- Chr02 2 2277418 4554834
- Chr02 3 4554835 6832251
- Chr02 4 6832252 9109668
- Chr03 1 1 2277417
- Chr03 2 2277418 4554834

Once chunk size is optimized, jobs (both GATK’s “CombineGVCFs” and “GenotypeGVCFs” functions) can be distributed and parallelized by chunks (Additional file 2: Fig. S1f–g). Leveraging this algorithm ensures that the creation of disjoint variant intervals is optimized based on genome size and computational resources, thereby preventing the underutilization of resources and the reduction of execution times.

### HPC-GVCW benchmarking

To evaluate the precision of SNP identification of GVCW, we initially assessed the workflow across three computational platforms — i.e., supercomputer, clusters, and high-end workstations, using a subset of The 3000 Rice Genome Project (3 K-RGP) dataset [6] (*n* = 30) mapped to The International Rice Genome Sequencing Project (IRGSP) Reference Sequence (RefSeq) [36]. We observed a 93.8–94.3% identical call rate across the three platforms and a 83–94% identical call rate when compared with previously published results [37] (Additional file 2: Fig. 2a).

**Fig. 2:**
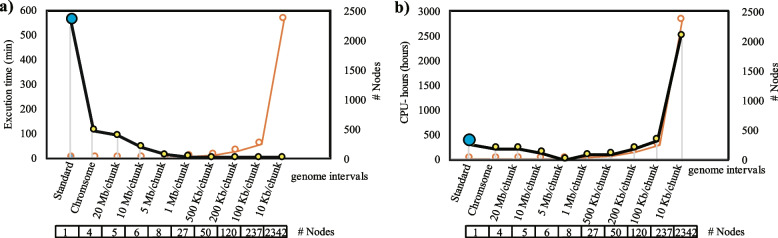
Benchmarking of the Phase 3 GIS parallelization HPC-GVCW as compare with the standard GATK pipeline using 30 resequenced rice accessions mapped to a single reference genome, **a** execution time and **b** CPU hours (execution time × number of nodes) for job completion. Notes: Comparisons were tested between the standard GATK pipeline without chunks using 1 node (blue dots), and HPC-GVCW using a range of computing nodes chunked length combinations, i.e., chunks sizes of 10 Kb, 100 Kb, 200 Kb, 500 Kb, 1 Mb, 5 Mb, 10 Mb, 20 Mb, and chromosome level, which use 2342, 237, 120, 50, 27, 8, 6, 5, and 4 nodes, respectively (yellow dots)

Using the same data set of 30 resequenced rice accessions mapped to a single reference genome we compared execution times for both the standard non-parallelization protocol (i.e., GATK) and our genome chuck parallelization protocol (HPC-GVCW) for the “combining gvcfs” Phase 3 algorithm. GATK used 9.5 h (570 min) to complete, regardless of platform used (Fig. 2a). Using the GIS algorithm with different chunk sizes (i.e., 100 Kb, 200 Kb, 500 Kb, 1 Mb, 5 Mb, 10 Mb, 20 Mb, and whole chromosomes) and node combinations (i.e., 2342, 237, 120, 50, 27, 8, 6, 5, and 4), execution times spanned from a maximum of 112 min using whole chromosome chunks to 2 min with 10 kb chunks and 2342 nodes. Overall, the execution times were 5–283 times faster than the standard GATK non-parallelization method.

We further compared the efficiency of total CPU hours (i.e., the execution time if all jobs were operated between the standard and genome chunk strategies) between GATK and HPC-GVCW. For GATK, a total of 304 CPU hours was required (9.5 h × 1 node × 32 cores/node) (Fig. 2b), vs. HPC-GVCW which ranged from 63 (chunk size = 5 Mb, nodes = 8) to 2511 (chunk size = 10 Kb, nodes = 2342) hours when using different chunk size/node combinations (Fig. 2b). This equates to a maximum of 4.8 times more efficient, to 8 times less efficient as compared to the standard GATK approach, respectively. Of note, we found that the number of CPU hours either increased or decreased at chunk sizes greater or less than 5 Mb when using HPC-GVCW, which is recommended when using the workflow.

Overall, our results reveal that execution time can be reduced by a maximum of 283 times when the smallest genome interval is set to 10 Kb/chunk, and CPU efficiency could be improved 4.8 times using a genome interval set to 5 Mb/chunk for HPC-CVCW, as compared with GATK.

### HPC-GVCW benchmarking for multiple crop species

To test if HPC-GVCW could be widely used across multiple crop species, we re-called SNPs using previously published resequencing/reference genome data sets for rice, sorghum, maize, and soybean (Additional file 3: Table S1 and Availability of data and materials for details). Using KAUST’s Shaheen 2 supercomputer with 30 K cores, processing 3,024 resequenced samples (3 K-RGP) mapped to a single rice reference genome took 94 h (i.e., 3.91 days) (Additional file 3: Table S2). For the sorghum, maize, and soybean data sets, due to the small number of samples, we only benchmarked HPC-GVCW on a hybrid cluster with 3000 cores and found that even for a 2.4 Gb maize genome [38], SNP calling for 282 samples could be completed within ten days (Additional file 3: Table S2). Our benchmarking test identified 26.5 M, 32.7 M, 167.6 M, and 15.9 M SNPs for rice (IRGSP-1.0), sorghum (BTx623), maize (B73 v4), and soybean (Gmax 275 v2.0), respectively (Table 1 and Additional file 3: Table S3). To assess the accuracy of the SNP calls produced through HPC-GVCW compared with previous reports, we found that 86.3% of the rice (22.8 M) and 89.3% of the sorghum (29.2 M) SNPs were identical (Additional file 2: Fig. S2c–d and Additional file 3: Table S3). For maize, only 25% of the SNP calls overlapped which was likely due to the software and strategy used for SNP calling and filtering [39]. For soybean, a direct comparison was not possible due to lack of data availability.

**Table 1:** Number of SNPs identified across four major crop species using their most recent public genome releases

Species	Reference genome	Acronyms	GenBank ID	Number of SNPs	SNPs in exons	SNPs in 3′ UTR	SNPs in 5′ UTR	5′ UTR premature start codon gain variant	Missense variant	Start lost	Stop gained	Stop lost
Rice *(Oryza sativa)* Genome size: ~ 400 Mb	GJ-temp: IRGSP	IRGSP	GCF_001433935.1	26,516,112	3,060,410	319,632	232,847	29,622	1,461,451	3048	38,699	2958
	GJ-subtrp: CHAO MEO	CM	GCA_009831315.1	27,024,845	3,069,706	356,381	233,761	29,316	1,462,534	3037	38,571	3012
	GJ-trop1: Azucena	AZ	GCA_009830595.1	27,316,403	3,081,793	345,485	226,235	28,131	1,473,280	2984	38,824	2925
	GJ-trop2: KETAN NANGKA	KN	GCA_009831275.1	27,331,337	3,031,741	335,086	219,831	27,464	1,448,804	3052	38,543	3048
	cB: ARC 10497	ARC	GCA_009831255.1	27,286,525	2,984,499	324,769	211,937	26,277	1,425,562	2965	37,501	2984
	XI-1A: ZhenShan97RS3	ZS97	GCA_001623345.2	27,439,649	3,504,390	573,128	406,815	53,607	1,664,226	3322	42,456	3344
	XI-1B1: IR 64	IR64	GCA_009914875.1	27,084,312	2,822,657	311,142	203,724	25,188	1,342,849	2618	34,958	2729
	XI-1B2: PR 106	PR106	GCA_009831045.1	27,461,145	3,029,730	343,797	224,081	27,840	1,443,799	2901	37,805	2926
	XI-2A: GOBOL SAIL	GS	GCA_009831025.1	27,608,213	2,885,485	293,846	198,221	24,849	1,388,477	2867	36,840	2909
	XI-2B: LARHA MUGAD	LM	GCA_009831355.1	27,974,114	2,921,223	307,604	206,271	25,723	1,402,841	2870	37,200	2961
	XI-3A: LIMA	LIMA	GCA_009829395.1	27,053,048	2,838,843	301,480	197,894	24,453	1,360,673	2839	36,103	2867
	XI-3B1: KHAO YAI GUANG	KYG	GCA_009831295.1	27,378,477	2,911,252	307,567	201,613	24,948	1,394,212	2840	36,680	2840
	XI-3B2: LIU XU	LX	GCA_009829375.1	27,759,204	2,939,867	311,835	213,624	26,747	1,411,721	2943	37,483	3052
	XI-adm: MH63RS3	MH63	GCA_001623365.2	27,503,492	3,509,396	603,812	422,385	55,137	1,661,569	3306	41,928	3370
	cA1: N22	N22	GCA_001952365.3	27,594,493	3,019,972	328,996	229,046	28,380	1,443,123	2931	37,919	2985
	cA2: NATEL BORO	NABO	GCA_009831335.1	28,044,207	2,979,119	312,640	212,806	26,394	1,433,853	2976	38,230	3075
Sorghum (*Sorghum bicolor*) (Genome size: ~ 600 Mb)	BT623v3.1	-	GCF_000003195.3	32,698,281	1,078,742	793,513	675,414	96,219	593,563	1442	13,124	1349
	Tx2783	-	GCA_903166285.1	32,537,001	752,298	327,512	205,336	25,181	434,942	888	15,706	7822
	Tx436	-	GCA_903166325.1	32,748,001	868,964	422,070	247,710	30,256	503,717	917	17,873	9090
	Tx430	-	GCA_003482435.1	35,102,930	1,194,497	360,556	236,007	28,860	656,657	1527	15,144	1788
Maize (*Zea mays*) Genome size: ~ 2000 Mb	B73v4	-	GCF_000005005.2	167,604,407	5,789,626	3,758,096	3,413,940	510,132	3,115,092	6621	122,988	6559
	B73v5	-	GCA_902167145.1	170,004,877	3,073,808	1,325,232	1,023,768	130,747	1,670,295	3461	58,418	3864
	Mo17v2	-	GCA_022117705.1	172,357,693	2,070,795	285,667	184,521	21,221	1,156,699	2433	47,826	2713
Soybean (*Glycine max*) Genome size: ~ 1000 Mb	Wm82.a2.v1	-	Gmax 275	15,994,704	812,611	267,541	194,096	25,424	500,153	714	14,282	1003
	JD17	-	GCA_021733175.1	16,341,705	569,416	213,129	147,393	18,852	335,286	808	10,107	1196

### HPC-GVCW at production scale — a 25-genome SNP dataset for multiple crop species

Since the majority of publicly available SNP data for major crop species have yet to be updated on the recent wave of ultra-high-quality reference genomes coming online, we applied HPC-GVCW to call SNPs, with the identical large resequencing datasets, on the most current and publicly available genome releases for rice (i.e., the 16 genome Rice Population Reference Panel) [15, 40, 41], maize (B73 v4, B73 v5, and Mo17v2) [16, 42], sorghum (Tx2783, Tx436, and TX430) [43], and soybean (Wm82 and JD17) [44].

As a result, a total of 1.1 billion SNPs were identified across the 25-genome data set, including 438.4 million SNPs based on a subpopulation-aware 16-genome rice reference panel (RPRP, avg. 27.3 M/reference), 133.1 million SNPs for 4 sorghum reference genomes (avg. 32.6 M/reference), 509.9 million SNPs for 3 maize reference genomes (avg. 169.9 M/reference), and 32.3 million SNPs for 2 soybean reference genomes (avg. 16.2 M/reference) (Table 1). Of these, 1.67–16.49% (0.93–12.96 M SNPs) and 0.71–6.44% (0.37–3.76 M SNPs) of total SNPs were predicted (with SNPEff [45]) to fall within and around genes, and their effects on genes, respectively (Table 1 and Additional file 3: Table S4).

### Novel SNPs in rice

Having the ability to map large-scale resequencing datasets rapidly (e.g., 3 K-RGP) to multiple genomes (e.g., the 16-genome RPRP dataset), HPC-GVCW opens the possibility to discover and rigorously interrogate population-level pan-genome datasets on multiple scales — i.e., pan-genome, genome and single gene scale.

#### Pan-genome scale

**Fig. 3:**
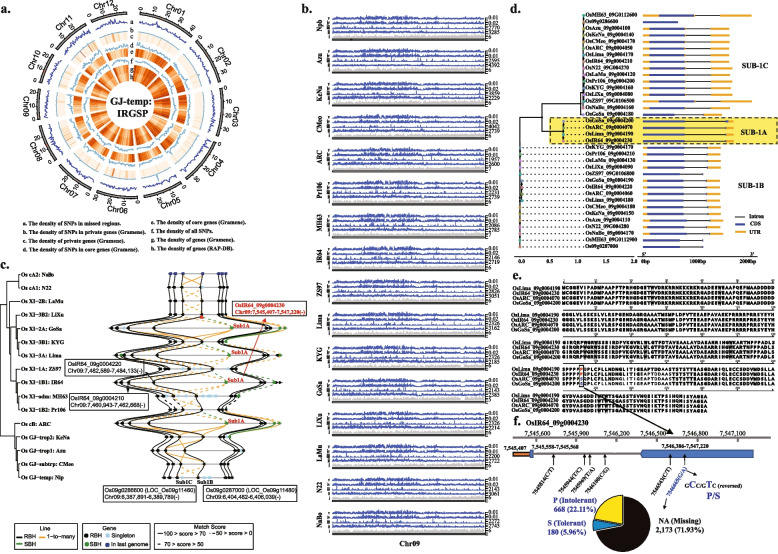
Rice Population Reference Panel (RPRP) [15] pan-genome variant analysis. **a** Circos plot depicts the distribution of genomic attributes along the IRGSP RefSeq (window size = 500 Kb). **b** Comparison of genomic attributes, i.e., genes, SNPs, *Pi*, and *Theta* on chromosome 9 across the 16 RPRP pan-genome data sets (window size = 10 Kb). **c** Rice Gene Index (RGI) comparison of the *Sub* loci across the 16 RPRP pan-genome data set. **d** Phylogenetic analysis of *Sub1A*, *Sub1B*, and *Sub1C* across the 16 RPRP pan-genome data set. **e** Amino acid alignment of the *Sub1A* gene across the RPRP. **f** Survey of SNPs within the *Sub1A* gene across the 3 K-RGP resequencing data set. This analysis revealed the genomic status of the *Sub1A* gene (presence/absence; submergence tolerance/intolerance) across the 3 K-RGP data set

Our analysis of the 3 K-RGP dataset [6] mapped to the 16-genome RPRP dataset [15] revealed a core genome of 314.1 Mb, an average dispensable genome of 56.55 Mb, and a private genome of ~ 745 Kb/genome (see Methods for definitions), that contain ~ 22.4 M, 3.2 M and 33.8 K SNPs, respectively (Additional file 2: Fig. S3, and Additional file 3: Table S5). We found that an average of 36.5 Mb of genomic sequence is absent in a single rice genome but is present in at least one of the other 15 RPRP data sets, which is equivalent to ~ 2.1 M SNPs (Fig. 3, Additional file 2: Fig. S3, and Additional file 3: Table S5). For example, when considering the flagship reference genome for rice, i.e., the IRGSP RefSeq [36], a total of ~ 36.6 Mb of genomic sequence is completely absent in the IRGSP RefSeq but is found spread across at least one of the 15 genomes (~ 2.43 Mb/genome), and includes ~ 2.3 M previously unidentified novel SNPs (Fig. 3, Additional file 3: Table S5).

Performing a similar analysis on gene content using the subpopulation-aware Rice Gene Index (RGI) [41] enabled us to identify an average of 24,700, 6577, and 293 core, dispensable and private homologous gene groups (see Methods for definitions) across the 16-genome RPRP data set, respectively (Additional file 3: Table S5), equating to 5.5 M SNPs (2.4 M exonic), 0.8 M SNPs (0.2 M exonic), and 37.8 K SNPs (9.6 K exonic) (Fig. 3, Additional file 2: Fig. S4 and Additional file 3: Table S5), respectively. Importantly, on average, a total of ~ 10.3 K genes present in 15 of the 16 RPRP genomes (687 genes/genome) are absent in a single RPRP genome, and equates to ~ 1.4 M SNPs (Fig. 3, Additional file 2: Fig. S4 and Additional file 3: Table S5). Again, taking the IRGSP RefSeq as an example, a total of 9812 genes detected across the rice pan-genome reference panel (RPRP) of 15 genomes are absent in the IRGSP RefSeq. Across these genes lie 1.3 M novel SNPs [46], of which 19.22% (i.e., 248,403) are predicted to have impacts on gene function (i.e., 4537 5′ UTR premature start codon gain variants; 229,184 missense variants; 1519 stop codon lost variants; 11,869 stop gained variants; and, 1294 stop lost variants**)** (Fig. 3 and Additional file 3: Table S6).

To validate these potentially functional SNPs, we measured the frequency of all 248,403 SNPs across the 3 K-RGP data set as shown in Additional file 2: Fig. S5a. The results show that 76.31% (189,564) of these putative functional SNPs could be identified within three or more rice accessions, thereby confirming the presence and quality of these SNP variants. These results show that much of the collective rice pan-genomes remain to be explored for crop improvement and basic research.

#### Genome scale — Zhenshan 97 (ZS97)

Open chromatin regions (OCRs) are special regions of the genome that can be accessed by DNA regulatory elements [47, 48]. Chromatin accessibility (CA) of OCRs can affect gene expression, epigenetic modifications, and patterns of meiotic recombination of tissue cells that could lead to important regulatory effects on biology observation [49, 50]. For rice, using the IRGSP RefSeq and the 3 K-RGP dataset, we previously annotated 5.06 M variants that were located in OCRs, of which ∼2.8% (~ 142,000) were classified as high-impact regulatory variants that may play regulatory roles across multiple tissues [51].

To search for novel SNPs in OCRs that are not present in the IRGSP RefSeq, we scanned for SNPs in OCRs of ZS97, a *Xian/Indica* variety, as a test case. First, our analysis revealed that approximately 14.6% of the ZS97 genome contains OCRs across the 6 tissues investigated, i.e., flag leaf, flower, lemma, panicle, root, and young leaf (Additional file 2: Fig. S6). We then conducted an intersection analysis of identified OCRs (peak regions) with variant call format (VCF) files and discovered 3,303,820 SNPs located within OCRs of the ZS97 genome (Additional file 3: Table S7), of which 7,441 were novel (i.e., relative to the IRGSP RefSeq). This equates to 6.23% of the 1.19 M ZS97 novel SNPs discussed above (Additional file 3: Table S7).

To validate these SNP, we again measured SNP frequency across 3 K-RGP for all 7441 novel SNPs and found that 78.13% (5814) of these SNPs could be identified in three or more accessions (Additional file 2: Fig. S5b).

To assess the potential functional impact of these novel ZS97 SNPs, we established thresholds by selecting the top and bottom five percent of variation scores from previously scored SNP variation data across as reported [51], which led to the identification of 855 SNPs (Additional file 3: Table S6). Notably, these SNPs accounted for approximately 33.3% of the loci with significant variations, which are considered large-effect SNP loci with a greater impact on chromatin accessibility (CA). These results indicate the effects of physical access to chromatinized DNA, to binding, allowing for active gene transcription for novel SNPs.

#### Single gene scale — Sub1A

Many of the genes and SNPs identified in our pan-genome variant analysis have yet to be tested for their contributions to agronomic performance and biotic and abiotic stresses. For example, prolonged submergence during floods can cause significant constraints to rice production resulting in millions of dollars of lost farmer income [52]. One solution to flooding survival has been to cross the *Sub1A* gene, first discovered in a tolerant *indica* derivative of the FR13A cultivar (IR40931-26) in 2006 [52], into mega rice varieties such as Swarna, Sambha Mahsuri, and IR64 [52, 53]. Our analysis of the *Sub1A* locus across the pan-genome of rice showed that this gene could only be observed in 4 out of 16 genomes in the RPRP data set, including IR64 (Fig. 3c, d). Since *Sub1A* is absent in the IRGSP RefSeq, the genetic diversity of this locus can only be revealed through the analyses of reference genomes that contain this gene. Thus, we applied the IR64 reference as the base genome for SNP comparisons, and identified a total of 26 SNPs in the *Sub1A* locus across 3 K-RGP, 6 of which have minor allele frequencies (MAF) greater than 1% (Fig. 1F), including a previously reported SNP (7,546,665-G/A), which is also validated by 4 gene sequences, i.e., OsIR64_09g0004230, OsLima_09g0004190, OsGoSa_09g0004200, and OsARC_09g0004070. This variation resulted in a non-conservative amino acid change from serine (S, *Sub1A-1*, tolerance-specific allele) to proline (P, *Sub1A-2*, intolerance-specific allele) [52] (Fig. 3e, f). The majority of accessions in the 3 K-RGP data set (i.e., 2173) do not contain the *Sub1A* gene, while 848 do, 668 of which (22.11%) have the *Sub1A-2* allele, while 180 accessions (5.96%) contain the *Sub1A-1* allele (Fig. 3f). Understanding the genetic diversity of the *Sub-1A* gene at the population level helps us understand and filter variants that are predicted to show flooding tolerance across the 3 K-RGP, which could be further applied to precise molecular-assisted selection (MAS) breeding programs. In addition, such pan-genome analyses may also reveal new variants that could provide valuable insights into the molecular mechanisms of flooding tolerance.

## Discussion

With the ability to produce ultra-high-quality reference genomes and population-level resequencing data — at will — accelerated and parallel data processing methods must be developed to efficiently call genetic variation at scale. We developed a publicly available open-source high-performance (CPU-based) computing pipeline (HPC-GVCW) that is supported across diversified computational platforms, i.e., desktops, workstations, clusters, and other high-performance computing architectures. In addition, HPC-GVCW was containerized for both Docker [54] and Singularity [55] for reproducible results without reinstallation and software version incompatibilities.

Comparison of SNP calls on identical data sets (i.e., rice 3 K-RGP to the IRGSP RefSeq and 400 samples from Sorghum Association Panel to the BT623v3.1) yielded similar results, however, run times could be reduced from more than six months to less than one week, as in the case for rice 3 K-RGP [6]. The GVCW pipeline enabled the rapid identification of a large amount of genetic variation across multiple crops, including sorghum, maize, and soybean on the world’s most up-to-date, high-quality reference genomes. These SNPs provide an updated resource of genetic diversity that can be utilized for both crop improvement and basic research, and are freely available through the SNP-Seek [56], Gramene web portals [57], and KAUST Research Repository (KRR [58]).

Key to our ability to rapidly call SNPs on a variety of computational architectures lies in the design of the HPC environment and the distribution of work across multiple nodes. Our next steps will be to apply GVCW on improved computing platforms, e.g., KAUST Shaheen III with unlimited storage and file numbers, 5000 nodes, faster input and output (I/O), and tests on larger forthcoming data sets [59]. In addition to GATK, other SNP detection strategies such as the machine learning-based tool “DeepVariant” [3], which shows better performance in execution times with human data [5], have yet to be widely used in plants. With a preliminary analysis of the rice 3 K-RGP dataset, “DeepVariant” identified a larger number of variants at a similar or lower error rate compared to GATK [60]. To test how artificial intelligence (AI) can be used to improve food security by accelerating the genetic improvement of major crop species, we plan to integrate “DeepVariant” into our HPC workflow to discover and explore new uncharacterized variation. In addition, we also plan to apply similar pan-genome strategies on more species beyond rice, sorghum, maize, and soybean to discover and characterize hidden SNPs and diversity, which could provide robust and vital resources to facilitate future genetic studies and breeding programs.

## Conclusions

We developed HPC-GVCW for variant calling in major crops, which can reduce execution times > 280 fold, as well as increase efficiency > 4.8 fold as compared with the GATK ‘best practice’ workflow [19]. A new algorithm (“Genome Index splitter”) for running ‘CombineGVCFs’ was designed to parallelize this step and was found to be 19 times faster than available default options. We demonstrated that the entire workflow can be used on a variety of computing platforms, such as hybrid clusters, and high-end workstations using Docker and Singularity images. Using HPC-GVCW, we called population panel variants for the latest high-quality genome references and created 25 immediately applicable datasets with an average of 27.3 M, 32.6 M, 169.9 M, and 16.2 M SNPs for rice (16 population panel references), sorghum (4), maize (3), and soybean (2), respectively. Analysis of a 16-genome rice reference panel revealed ~ 2.3 M novel SNPs relative to the IRGSP RefSeq, which equates to an approximate 8% overall increase in SNP discovery that can be applied immediately to precise molecular-assisted selection (MAS) breeding programs and functional analyses.

## Methods

### SNP identification workflow

The SNP identification workflow presented here (i.e., genome variant calling workflow (GVCW)) was developed to provide a freely available and containerized high-performance computational platform to run the Genome Analysis Toolkit (GATK) “best practice” software (https://gatk.broadinstitute.org/hc/en-us/sections/360007226651-Best-Practices-Workflows) for the analysis of large resequencing data sets mapped to multiple reference genomes (see Additional file 1 for a detailed description). Briefly, genome resequencing data from multiple crop species was used for quality control, mapping, SNP calling, and multi-sample joint genotyping. Raw Illumina read data was scanned by Fastqc (0.11.8) [61], and trimmed with Trimmomatic (v0.38) [62] with the following parameters: “ILLUMINACLIP: TruSeq3-PE-2.fa: 2:30:10 LEADING: 3 TRAILING: 3 SLIDINGWINDOW: 4:15 MINLEN: 36.” Trimmed reads were then aligned to their respective high-quality reference genome sequences using Burrows-Wheeler Alignment (BWA-MEM, v0.7.17) [63] under default parameters. Mapped reads with quality scores ≥ 30 were then sorted using SAMTools [64] (v1.8). Duplicate reads were marked and re-grouped using GATK’s (v4.1.6) [18] “MarkDuplicates” and “AddOrReplaceReadGroups” functions. SNPs for each accession (gVCF) were called using the GATK’s HaplotypeCaller [19]. GATK functions “CombineGVCFs” and “GenotypeGVCFs” were then used for joint genotyping to produce merged VCFs from gVCFs for each sample by intervals. Finally, SNPs were extracted from the joint genotypes using GATK’s “SelectVariants” and “VariantFiltration” functions with the following parameters: “QUAL < 30.0 || QD < 2.0 || MQ < 20.0 || MQRankSum < -3.0 || ReadPosRanKSum < -3.0 || DP < 5.0” to filter for high-quality of SNPs.

### Sequence data

Twenty-five reference genome sequences, including the latest gap or near gap-free assemblies of rice, sorghum, maize, and soybean, are listed in Table 1. All resequencing data was downloaded from the following public databases: rice 3 K-RGP data set (3,024 samples) [6]; sorghum association panel (SAP, 400 samples) [10], maize association mapping panel (AMP, 282 samples) [39], soybean mini-core collection (MCC, 198 samples) [9] (Additional file 3: Table S3 and Data Availability).

### SNP annotation

SNPs located in coding regions across the 25 reference genome data sets were identified using their respective annotation files, and functional SNPs were predicted using SnpEff (v5.0e) [45].

### SNP visualization for rice and sorghum

SNP data for rice (i.e., ARC, N22, AZU, IR64, IRGSP, MH63 ZS97) and sorghum (Tx2783) genome data sets can be visualized at the following web portals, respectively:

- Rice: https://oryza.gramene.org/ (Gramene release 6, https://oryza.gramene.org/News).
- Sorghum: https://sorghumbase.org/ (Sorghumbase Release 6, https://www.sorghumbase.org/relnotes). Instructions for visualization can be found in Additional file 4. Two examples of putative SNPs that result in premature stop codons are shown in Additional file 2: Fig. S7.

### Structural variation (SV) update across the 16-genome rice population reference panel (RPRP)

In 2020, we published an index of large structure variations (> 50 bp, SVs) across the 16-genome RPRP that included the MH63RS2 and ZS97RS2 genome assemblies [40]. Here, we updated this index using the latest gap-free genome assemblies for these genomes — i.e., MH63RS3 and ZS97RS3 [65] — using the same methods as previously described. To validate this updated SV index, we randomly selected 50 insertions and 50 deletions across the 16 rice genome (RPRP), using the IRGSP RefSeq as the reference and the remaining 15 rice genomes as queries, which included a total of 1,500 entries ((50 + 50) × 15 = 1500).

We then manually validated each SV with alignment information in the Integrative Genomics Viewer (IGV) using raw reads and alignment blocks with Nucmer [66]. SVs were considered valid if the two methods could identify the identical insertion or deletion and resulted in 94.6% of the insertions and 99.3% of the deletions being validated as true SVs.

### Homologous gene identification across the 16-genome Rice Population Reference Panel (RPRP) based on sequence alignment and syntenic position

As with SVs above, we also updated our rice gene index (RGI) using updated MH63RS3 and ZS97RS3 gene annotations with identical pipeline [41]. Briefly, homologous gene sets across the 16-genome RPRP were identified using GeneTribe software [67], by combining protein sequence similarity and collinearity (i.e., synteny) information. Homologous relationships included “reciprocal best hits” (RBHs), “single-side best hits” (SBHs), one-to-many, and singletons. Based on the one-to-one relationships (both RBH and SBH), and considering the collinearity blocks, we removed redundant homologous gene groups to obtain 79,111 non-redundant homologous gene groups. Finally, these non-redundant homologous gene groups were clustered with the “Connected Graph Algorithm” [68] to obtain 41,137 homologous gene groups.

### Rice pan-genome SNP analysis

Using the updated SV and RGI data sets in combination with the 16-genome RPRP SNP data set, we conducted a pan-genome SNP analysis to classify genomic regions into core, dispensable, genome-specific, and genome-absent regions [69]. Core regions are defined as sequences that are present in all 16 RPRP genomes. Dispensable regions are defined as sequences that are observed in 2 to 15 of the 16 RPRP genomes. Genome-specific regions are defined as sequences that are present in only one of the 16 RPRP genomes, but absent in the remaining 15. Genome-absent regions are defined as sequences that are not present in one of the 16 RPRP genomes, but are present in at least one of the other 15 genomes. For the presence and absence of genes, we classified homologous gene groups as core, dispensable, specific, and absent genes, representing the same logic flow as large SVs. Bedtools (v2.30.0) [70] subcommand “subtract” was used for core region identification, and the subcommand “intersect” was used for SNP extraction.

### Chromatin accessibility of novel SNPs in open chromatin regions

Accessible Chromatin, combined with high-throughput sequencing (ATAC-seq) is widely used as one of the mainstream OCR detection methods [51, 71, 72]. In this study, ATAC-seq data from 6 tissues of ZS97RS3, i.e., flag leaf, flower, lemma, panicle, root, and young leaf were obtained from NCBI BioProject PRJNA705005 [73]. In the initial steps of analyzing raw ATAC-seq data, we conducted quality control using FastQC [74]. This quality control process involved evaluating the quality of sequenced bases, average GC content, and the presence of repetitive sequences. Notably, we observed variations in the content of the first four bases at the 5′ end of each sample. To address this issue, we further refined our data by using fastp (v0.12.4) [75] to remove low-quality data and trim 20 base pairs from the 5' ends. Subsequently, we employed BWA’s mem [76] algorithm to align the sequencing data with the ZS97RS3 rice genome while filtering out reads that mapped to mitochondrial and chloroplast DNA. Peak regions of open chromatin regions (OCRs) within the ATAC-seq data were identified using MACS2 [77] with specific parameters: “–shift -100 –extsize 200 –nomodel –B –SPMR -g 3.0e8 –call-summits -p 0.01.” Following this, peak call results from each individual sample were combined using BEDtools (v2.26.0) [70] with default settings for merging.

To assess the potential functional impact of novel SNPs, we employed the intragroup Basenji model to study their variation scores [51]. Based on the Basenji model training, we predict the effect of variation in different tissues on chromatin accessibility (CA) in neighboring genomic regions. For each variation, we construct two sequences that contain the mutation site and the sequences around it, differing only at the mutation site. We then predict CA in each of these two sequences and score the effect of variants by comparing the CA differences between the two genotypes in the 1 kb region around the mutation site. The higher the score of the SNP, the greater the effect on CA in open chromatin regions.

### Supplementary Information

**Additional file 1.** The Design and Performance of the High-Performance Computing-based Genome Variant Calling Workflow (HPC-GVCW).

**Additional file 2: Fig. S1.** GVCW data processing. a. Data pre-processing; b. Variant discovery; c. Chromosome split table for call set refinement; d. Conditions and prerequisites for chromosome split table creation; e. Optimal chunk size calculation; f. Parallel distribution across all the chromosomes for a given reference genome; g. Call set refinement; h. Variants to the table. **Fig. S2.** Venn diagrams show comparisons of SNP calls for different datasets, i.e., a and b. Rice (*n*=30); c. 3K-RGP full datasets for rice (*n*=3,024); d. Sorghum (*n*=400). **Fig. S3.** Large structural variation (> 50 bp) analysis of the 16-genome Rice Population Reference Panel (RPRP). a. Insertions, b. Deletions. **Fig. S4.** Circos plots depict the distribution of genomic attributes along the 12 chromosomes of the 16-genome RPRP data set (window size = 500 Kb). **Fig. S5.** Validation of (a) Novel Functional SNPs and (b) Novel SNPs in OCRs through the number of accessions where a SNP is present. **Fig. S6.** Histogram displaying the number of open chromatin regions (OCRs) identified in 6 tissues of ZS97. **Fig. S7.** SNP visualization of two putative SNPs that resulted in premature stop codons in sorghum (Tx2783) and rice (IR64). a. One C→T transition (Chr04, 6,047,465) for gene SbiRTX2783.04G076100 in the T2783 sorghum genome. b. One G→A transition (Chr01, 15,993) for gene OsIR64_010000010 in the IR64 rice genome.

**Additional file 3: Table S1.** Summary of workflow phases. **Table S2.** Performance of the genome variant calling workflow (w/GATK4) for rice, sorghum, maize and soybean. **Table S3.** Results of variant detection based on the automated workflow for rice, sorghum, maize, and soybean. **Table S4.** SNPEff annotations for rice, sorghum, maize, and soybean. **Table S5.** The number of SNPs was identified by using rice RPRP references. **Table S6.** Results of novel SNP annotation in genes. **Table S7.** Results of SNP analysis in open chromatin regions verse genome-wide regions.

**Additional file 4.** Step-by-step instructions of SNP visualization on Gramene panGenome GrameneOryza.

## Data Availability

All data generated or analyzed during this study are included in this published article, its supplementary information files, and publicly available repositories. HPC-GVCW is a fully open resource, with all scripts, workflows, and instructions available at Zenodo [78]. To enhance the flexibility of computing platforms and applications, robust containerization solutions, including Docker [54] and Singularity [55] were developed [79, 80]. All sequence data are available in public databases as follows. All genome assemblies for rice, sorghum, maize, and soybean were retrieved from NCBI (Table 1), except for Wm82.a2.v1, which is available at the Phytozome [81]. Genome resequencing data sets for rice (*n* = 3024) [6], sorghum (*n* = 400) [10], maize(*n* = 282) [39], and soybean (*n* = 198) [9] were retrieved from NCBI via BioProject accession numbers: PRJEB6180 [82], PRJEB50066 [83], PRJNA389800 [84], and PRJDB7281 [85] respectively. All SNP data produced for this 25-genome reference set have been publicly released through the SNP-Seek (https://snp-seek.irri.org/_download.zul), Gramene (http://ftp.gramene.org/collaborators/Yong_et_al_variation_dumps/), and KAUST Research Repository (KRR [58]) public databases for immediate access. In addition, SNP data for rice (i.e., ARC, N22, AZU, IR64, IRGSP, MH63 ZS97) and sorghum (Tx2783) genome data sets can be visualized at the Gramene (https://oryza.gramene.org/) and Sorghumbase (https://sorghumbase.org/) web portals, respectively (Additional file 4). Realignment data sets of near variant regions (cram file format) of the *O. sativa* 16-genome RPRP data set are available through Amazon Web Services (AWS) 3kricegenome bucket at SNP-Seek (https://snp-seek.irri.org/_download.zul). SNP datasets for sorghum, soybean, and maize are released at Gramene (http://ftp.gramene.org/collaborators/Yong_et_al_variation_dumps/), and KAUST Research Repository (KRR [58]). SNP datasets for sorghum can be visualized from the Sorghumbase web portal (https://www.sorghumbase.org/).

## Abbreviations

SNPs: Single-nucleotide polymorphisms
GATK: Genome Analysis Toolkit
CNVs: Copy number variants
GPUs: Graphics processing units
FPGAs: Field-programmable gate arrays
HPC: High-performance computing
GVCW: Genome variant calling workflow
GIS: Genome Index Splitter
OCRs: Open chromatin regions
CST: Chromosome split table
CPUs: Central processing units
ChrName: Chromosome name
Chunk_no: Chunk number, unique integer per chromosome
Start: Chromosome starting position
End: Chromosome end position
3 K-RGP: The 3000 Rice Genome Project (3024 samples)
IRGSP: The International Rice Genome Sequencing Project
RefSeq: Reference sequence
RPRP: Rice Population Reference Panel
RGI: Rice Gene Index
UTR: Untranslated region
VCF: Variant call format
CA: Chromatin accessibility
KAUST: King Abdullah University of Science and Technology
KRR: KAUST Research Repository
BWA: Burrows-Wheeler Alignment
gVCF: Genomic variant call format
SAP: Sorghum Association Panel
AMP: Association mapping panel
SVs: Structural variants
IGV: Integrative Genomics Viewer
RBHs: Reciprocal best hits
SBHs: Single-side best hits
I/O: Input and output
AI: Artificial intelligence
