# Supplementary material for: A high-performance computational workflow to accelerate GATK SNP detection across a 25-genome dataset
Source: BMC Biol. 2024 Jan 25;22:13. doi: 10.1186/s12915-024-01820-5 (PMC10809545; doi:10.1186/s12915-024-01820-5)
Supplement: Supplementary file 1 — Additional file 1. The Design and Performance of the High-Performance Computing-based Genome Variant Calling Workflow (HPC-GVCW). [file 12915_2024_1820_MOESM1_ESM.docx]

**Additional file 1: The Design and Performance of the High-Performance Computing-based Genome Variant Calling Workflow (HPC-GVCW)**

# **1. Automated Genome Variant Calling Workflow (GVCW) Design**

The genome variant calling workflow used in this study was designed and automated for large-scale genomic data sets (e.g., 3,024 Rice Genomes Project (3K-RGP)[6]), to significantly reduce manual data management processes such as tracking multiple jobs and dependencies on high-performance computing platforms (HPC). Multiple bioinformatics tools were employed at various workflow phases (also called “job steps”), and the output of earlier job steps can become the input for the next job step, and so forth. In addition, workflow resource requirements, like the number of CPUs, memory size, use of temporary files, and Java heap/stack size, were considered during job scheduling and automation[32,33].

Our variant calling workflow was divided into 4 phases:

## **Phase 1: Data pre-processing**

Phase 1 was designed to map clean resequencing reads to a specified reference genome using the BWA-MEM (v0.7.17) aligner[63]. After alignment, the reads are filtered based on quality scores (≥30), and sorted using SAMTools[64]. Next, GATK is used to carry out the realignment of bam files, where all mate-pair information from different sequence reads (*e.g.*, from similar rice genome samples) are synchronized between each read and its mate pair using GATK’s “FixMateInformation.” Lastly, read duplicates are identified and condensed into a single new read-group using GATK’s “MarkDuplicates” and “AddOrReplaceReadGroups” functions. A summary of the data pre-processing workflow is described in Additional file 2: Fig. S1a.

## **Phase 2: Variant discovery**

Phase 2 was designed to call variants for each sample and generate gVCFs files. This phase comprises two major steps: first, multiple sorted input files are merged into a single BAM file and (re)sorted into a merged BAM file using SAMTools. Next, SNPs are called simultaneously via a local *de novo*-assembly of haplotypes in an active region using GATK’s “HaplotypeCaller,” where a single gVCF file will be generated per sample. The Phase 2 workflow is described in Additional file 2: Fig. S1b.

## **Phase3: Call set refinement**

Phase 3 was designed to merge all variants per sample (stored in a gVCF file from Phase 2) into non-redundant joint genotype files (stored as VCF files) by chunks in two steps. First, all gVCF files are merged using the GATK’s “CombineGVCFs” function for each chunk (Additional file 2: Fig. S1c-f). Next, multi-sample joint genotyping is performed based on the merged VCF file for each chunk using the GATK’s “GenotypeGVCFs” function.

Both GATK’s “CombineGVCFs” and “GenomicsDB” functions are recommended for sample merging. “GenomicDB” is limited in the number of samples can import at one time, and requires samples to be grouped into multiple batches to address this limitation (<https://gatk.broadinstitute.org/hc/en-us/articles/360056138571-GenomicsDBImport-usage-and-performance-guidelines)>. However, “CombineGVCFs” can combine multiple intervals all at one time, once without the need to build a data store (<https://gatk.broadinstitute.org/hc/en-us/articles/360035891051-GenomicsDB>) as recently demonstrated for 10,588 human samples successfully[34]. Hence, we used “CombineGVCFs” for data parallelization in our workflow.

GATK’s “CombineGVCFs” and “GenotypeGVCFs” functions were initially designed to be executed on a single core because of programming limitations[32]. Unfortunately, assembling genotypes across a large number of samples into a single file can take an extremely long time, and requires huge amounts of memory, especially for species with large genome sizes. To address this limitation, the latest version of GATK offers the variant intervals feature (also referred to as “chunks”) for both “CombineGVCFs” and “GenotypeGVCFs.” To improve the performance in our workflow, disjoint variant intervals (chunks) are used to run in parallel across a node cluster on HPC platforms. In this design, merging variants from multiple samples (“CombineGVCFs”) and joint genotyping ( “GenotypeGVCFs”) can proceed for each chunk simultaneously (Additional file 2: Fig. S1g).

An algorithm called “Genome Index Splitter” (GIS) (<https://github.com/IBEXCluster/Genome-Index-splitter)> was designed to optimize the size and number of genomics intervals. In our recent GIS release (<https://github.com/IBEXCluster/Genome-Index-splitter>, release 1.2), GIS was updated to use MPI-based data distribution. that was also applied successfully for sorghum, maize, and soybean. In addition, GIS has been tested to work across various platforms and different workflows for major crop species over a broad range of genome sizes (GS) - e.g., rice [GS=400Mb] (<https://github.com/IBEXCluster/Rice-Variant-Calling/>[)](https://github.com/IBEXCluster/Rice-Variant-Calling/tree/main/For_Hybrd_Cloud)), and wheat [GS=15Gb] (<https://github.com/IBEXCluster/Wheat-SNPCaller)>.

## **Phase4: Variant matrix**

Phase 4 was designed to generate a genome-wide joint genotype by assembling all disjoint variant intervals from Phase 3 by combining all chunks into a single file using GATK’s “GatherVcfs” option. Chromosome-based SNPs can then be converted into a variant table with “HapMap” format[35] for post-processing analyses (Additional file 2: Fig. S1h).

## **2. Workflow flexibility**

All software environments and workflow scripts have been “containerized,” so the various workflow phases can be effortlessly switched from one system architecture to another (see code availability). For example, data preprocessing of many samples concurrently can be executed in a cluster computing environment (i.e., Phase 1 & 2). The user can then move the results from the cluster to an HPC system for Phase 3 - i.e., “Call set refinement” - without any changes in the existing workflow scripts. This flexibility offers more opportunities to collaborate and utilizes the various computing resources at different organizations. Further, users may migrate workflow environments into any computing platform, including a laptop, cluster computing, and supercomputers, because the software workflow environments are seamlessly supported for any target platform, as illustrated Fig. 1.
