# Supplementary material for: A high-performance computational workflow to accelerate GATK SNP detection across a 25-genome dataset
Source: BMC Biol. 2024 Jan 25;22:13. doi: 10.1186/s12915-024-01820-5 (PMC10809545; doi:10.1186/s12915-024-01820-5)
Supplement: Supplementary file 2 — Additional file 2: Fig. S1. GVCW data processing. a. Data pre-processing; b. Variant discovery; c. Chromosome split table for call set refinement; d. Conditions and prerequisites for chromosome split table creation; e. Optimal chunk size calculation; f. Parallel distribution across all the chromosomes for a given reference genome; g. Call set refinement; h. Variants to the table. Fig. S2. Venn diagrams show comparisons of SNP calls for different datasets, i.e., a and b. Rice (n=30); c. 3K-RGP full datasets for rice (n=3,024); d. Sorghum (n=400). Fig. S3. Large structural variation (> 50 bp) analysis of the 16-genome Rice Population Reference Panel (RPRP). a. Insertions, b. Deletions. Fig. S4. Circos plots depict the distribution of genomic attributes along the 12 chromosomes of the 16-genome RPRP data set (window size = 500 Kb). Fig. S5. Validation of (a) Novel Functional SNPs and (b) Novel SNPs in OCRs through the number of accessions where a SNP is present. Fig. S6. Histogram displaying the number of open chromatin regions (OCRs) identified in 6 tissues of ZS97. Fig. S7. SNP visualization of two putative SNPs that resulted in premature stop codons in sorghum (Tx2783) and rice (IR64). a. One C→T transition (Chr04, 6,047,465) for gene SbiRTX2783.04G076100 in the T2783 sorghum genome. b. One G→A transition (Chr01, 15,993) for gene OsIR64_010000010 in the IR64 rice genome. [file 12915_2024_1820_MOESM2_ESM.pdf]

Additional file 2: Supplementary Figures 1-7.

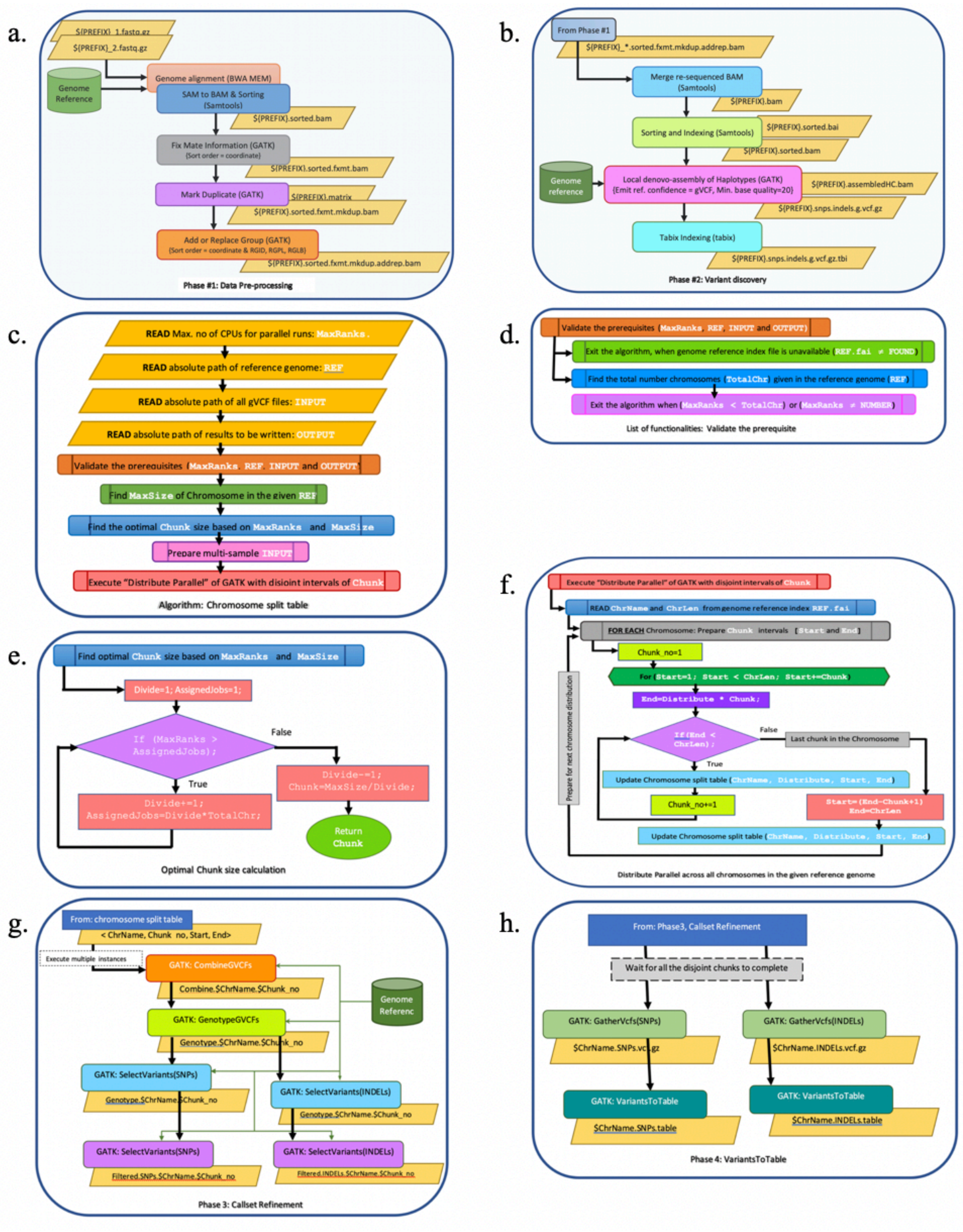

Fig. S1 GVCW data processing. a. Data pre-processing; b. Variant discovery; c. Chromosome split table for call set refinement; d. Conditions and prerequisites for chromosome split table creation; e. Optimal chunk size calculation; f. Parallel distribution across all the chromosomes for a given reference genome; g. Call set refinement; h. Variants to the table.

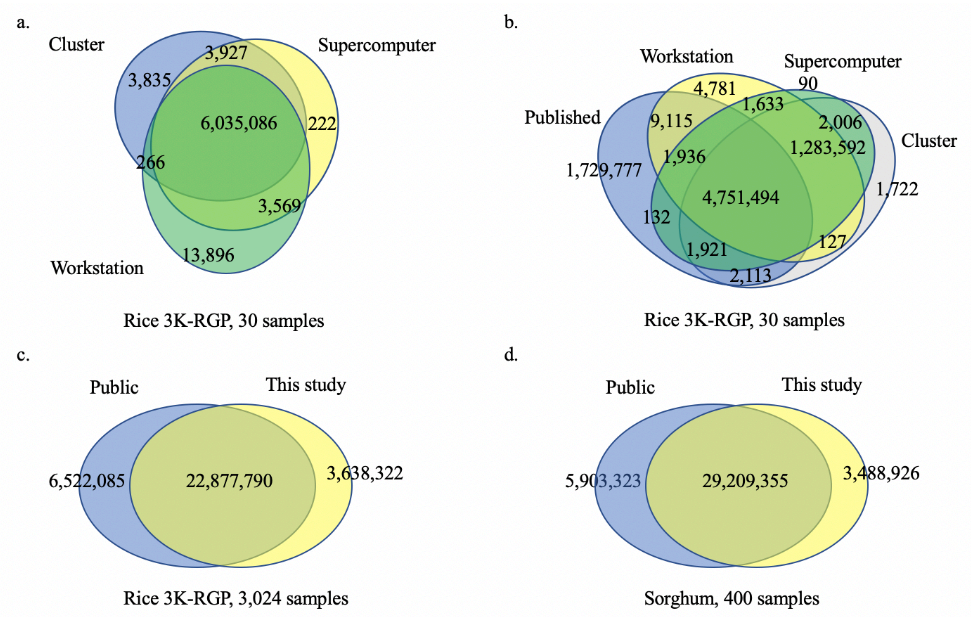

Fig. S2 Venn diagrams show comparisons of SNP calls for different datasets, i.e., a and b. Rice (n=30); c. 3K-RGP full datasets for rice (n=3,024); d. Sorghum (n=400).

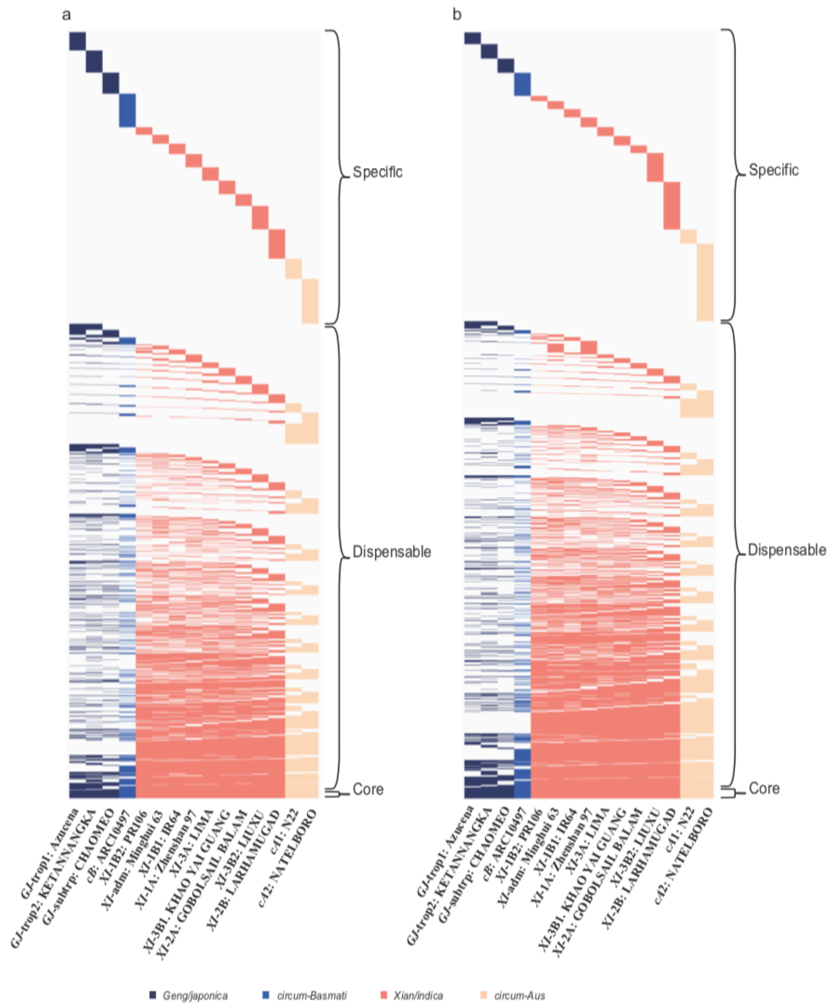

Fig. S3 Large structural variation (> 50 bp) analysis of the 16-genome Rice Population Reference Panel (RPRP). a. Insertions, b. Deletions.

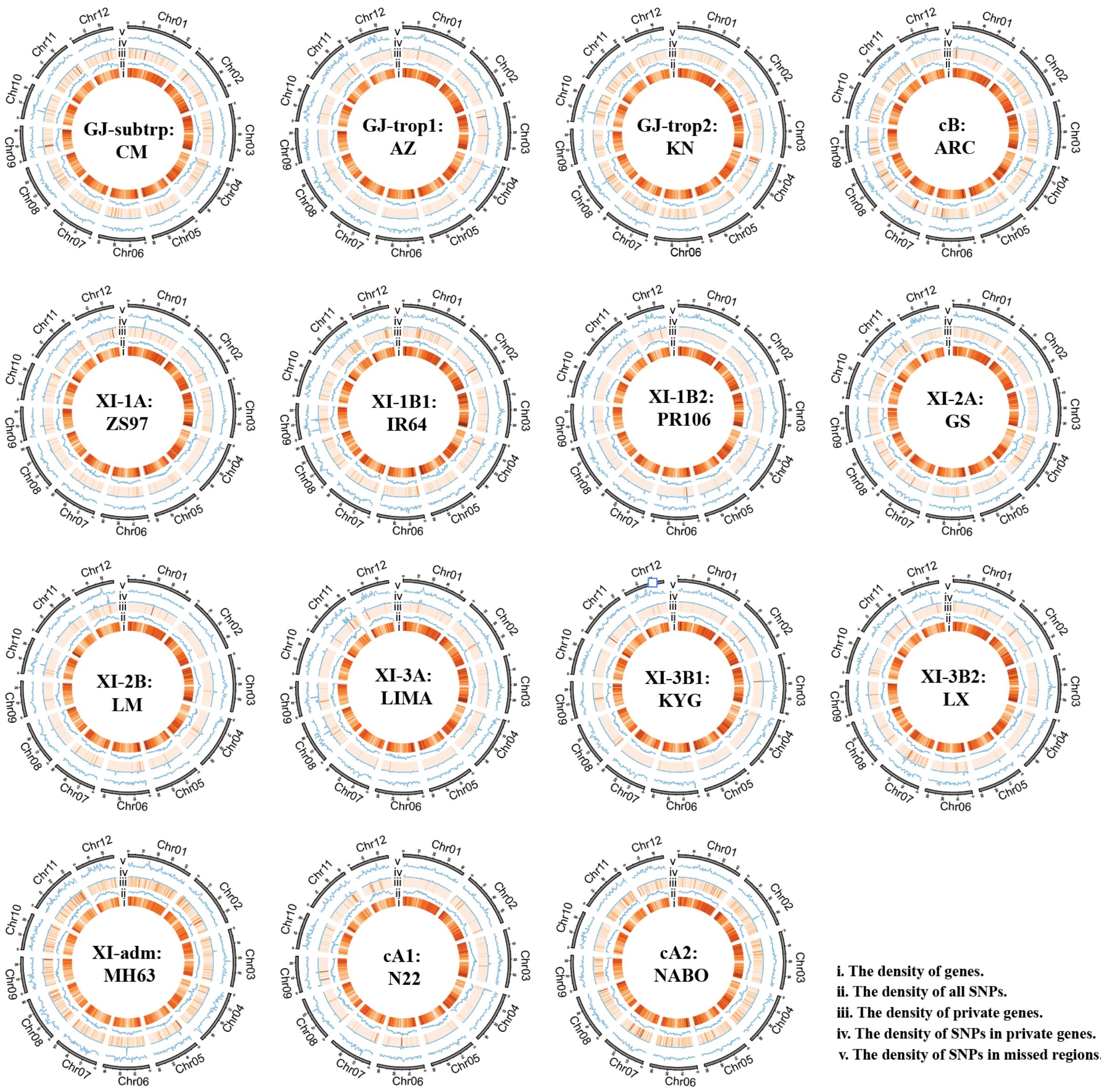

Fig. S4 Circos plots depict the distribution of genomic attributes along the 12 chromosomes of the 16-genome RPRP data set (window size = 500 Kb).

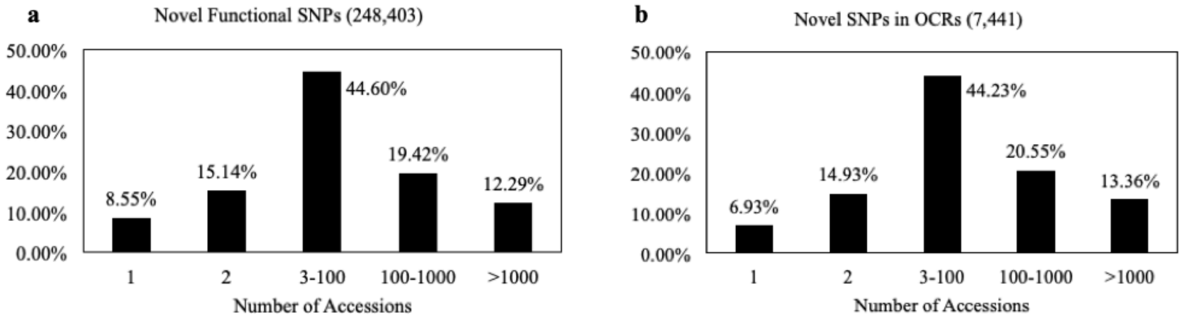

Fig. S5 Validation of (a) Novel Functional SNPs and (b) Novel SNPs in OCRs through the number of accessions where a SNP is present.

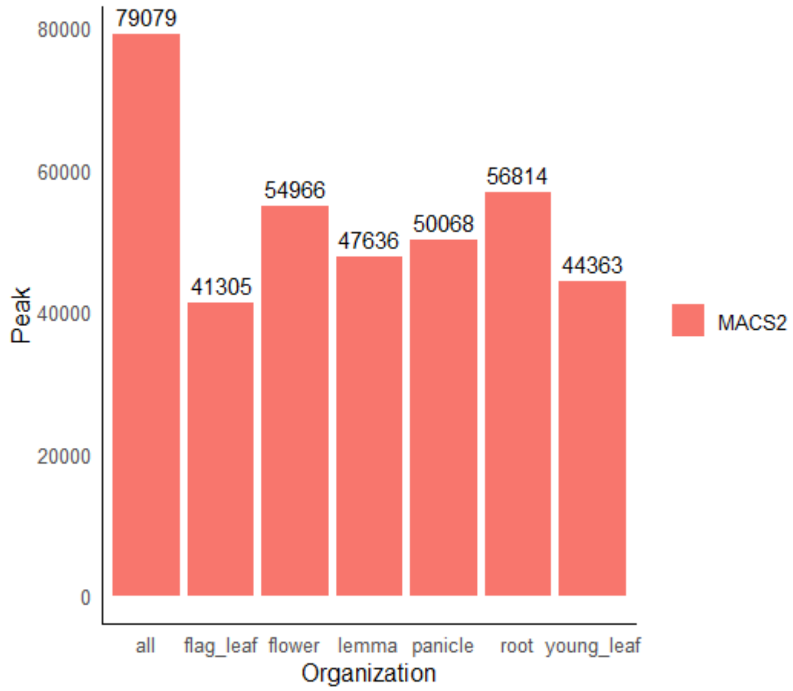

Fig. S6 Histogram displaying the number of open chromatin regions (OCRs) identified in 6 tissues of ZS97.

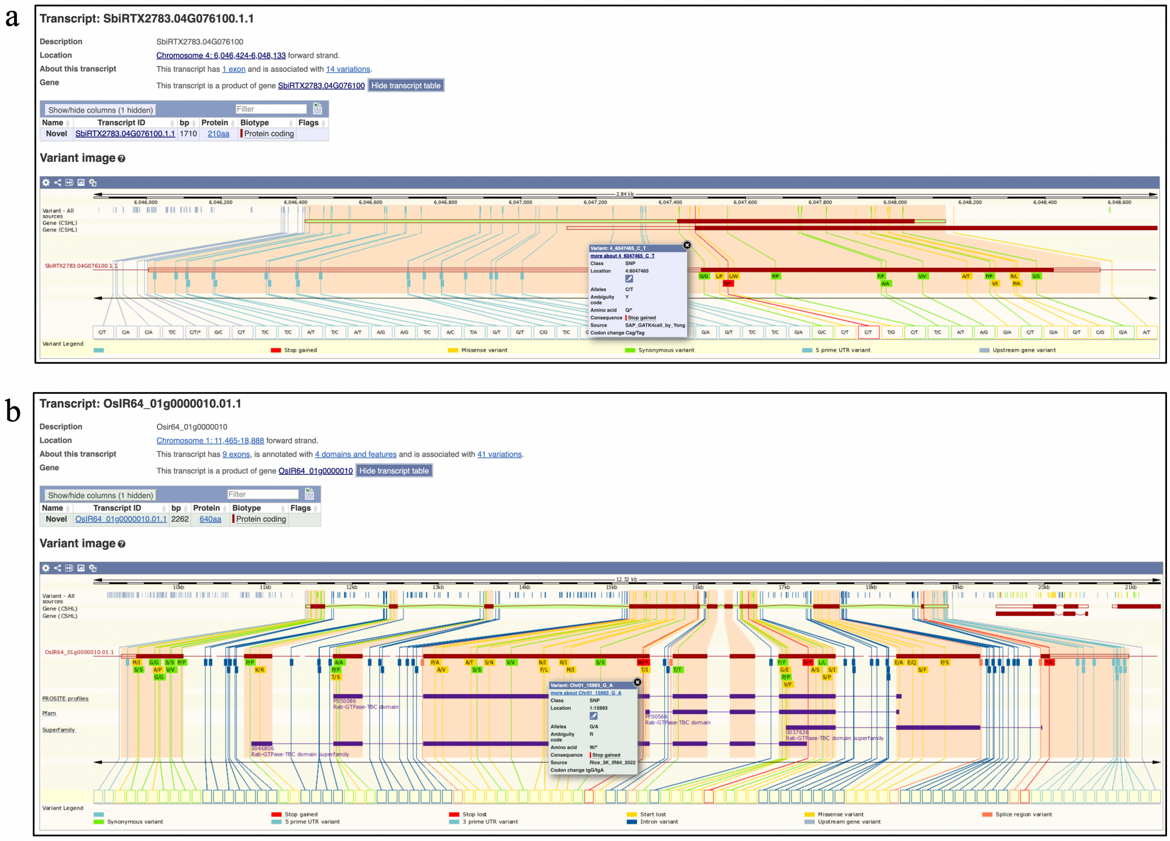

Fig. S7 SNP visualization of two putative SNPs that resulted in premature stop codons in sorghum (Tx2783) and rice (IR64). a. One C→T transition (Chr04, 6,047,465) for gene SbiRTX2783.04G076100 in the T2783 sorghum genome. b. One G→A transition (Chr01, 15,993) for gene OsIR64_010000010 in the IR64 rice genome.
