# Supplementary material for: A high-performance computational workflow to accelerate GATK SNP detection across a 25-genome dataset
Source: BMC Biol. 2024 Jan 25;22:13. doi: 10.1186/s12915-024-01820-5 (PMC10809545; doi:10.1186/s12915-024-01820-5)
Supplement: Supplementary file 4 — Additional file 4. Step-by-step instructions of SNP visualization on Gramene panGenome GrameneOryza. [file 12915_2024_1820_MOESM4_ESM.pdf]

Additional File 4: Step-by-step instructions of SNP visualization for subsite GrameneOryza.

SNP features are available on Gramene panGenome subsite GrameneOryza.

1. There are two ways to get to the GrameneOryza subsite homepage.

    1.1 from Gramene main website https://gramene.org/

        Click Plant Pan Genomes → Click Oryza subSite → Get to GrameneOryza

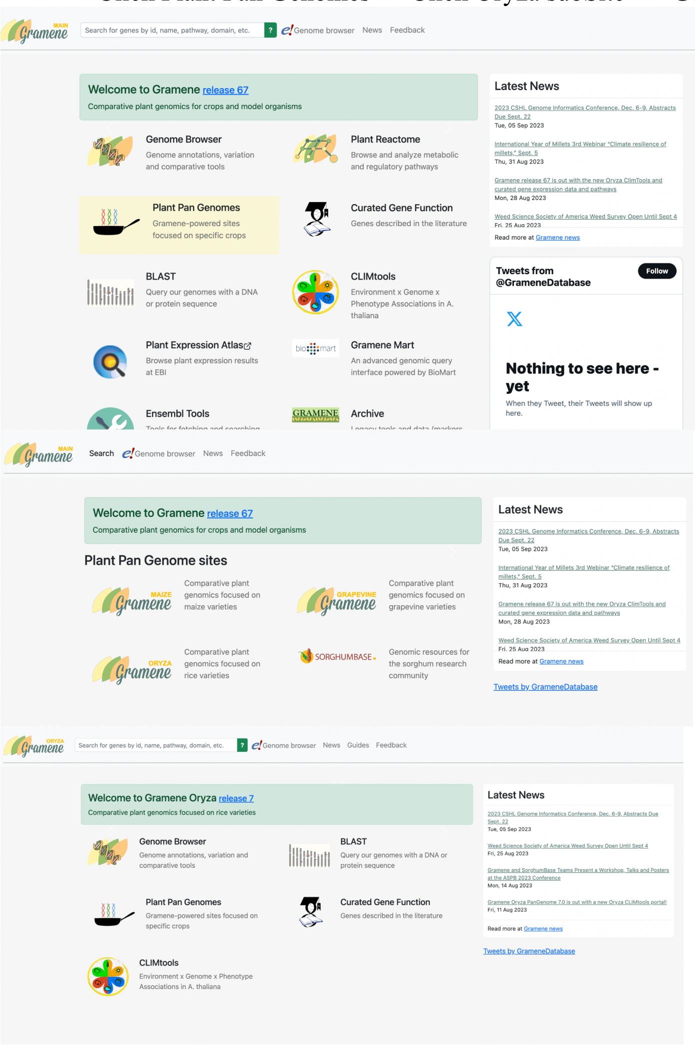

    1.2 Directly from url https://oryza.gramene.org/

2. There are at least three ways to access variation data. Currently there are 8 genomes with variation data available to view from the browser. They are

- Oryza sativa Japonica Nipponbare
- Oryza sativa indica XI-1B1 var. IR64
- Oryza sativa indica XI-adm var MH63
- Oryza sativa indica XI-1A var ZS97
- Oryza sativa japonica GJ-trop1 var. Azucena
- Oryza sativa circum-Basmati var. ARC 10497
- Oryza sativa Xian/Indica-3B2 var. Liu Xu
- Oryza sativa circum-Aus1 var. N22

    2.1 Directly land on a sample variation page through the genome homepage.

        Use Oryza sativa japonica GJ-trop1 var. Azucena as an example, from GrameneOryza subSite,

Click Genome Browser → open drop-down selection list of ‘Select a species’ and select ‘Oryza sativa japonica GJ-trop1 var. Azucena’ to land on the genome homepage → at genome homepage, click variation ‘sample variant’ icon → arrived at the variation page for this sample SNP

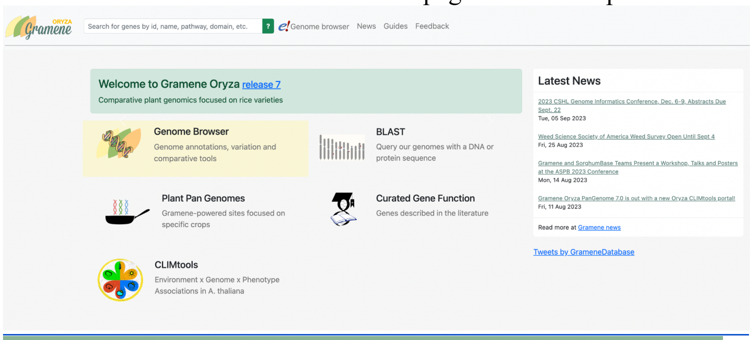

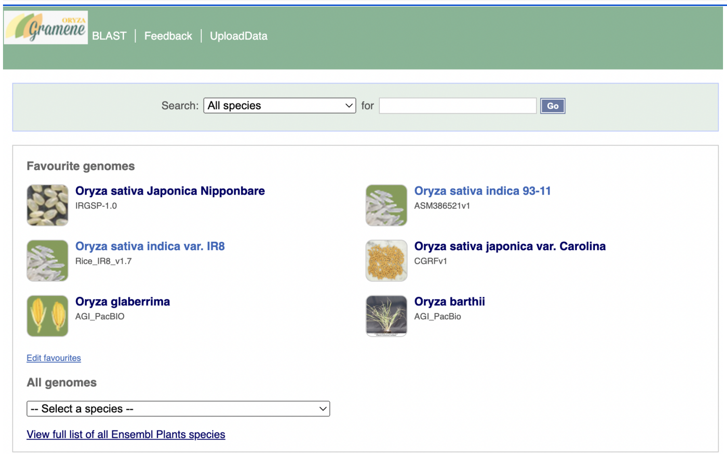

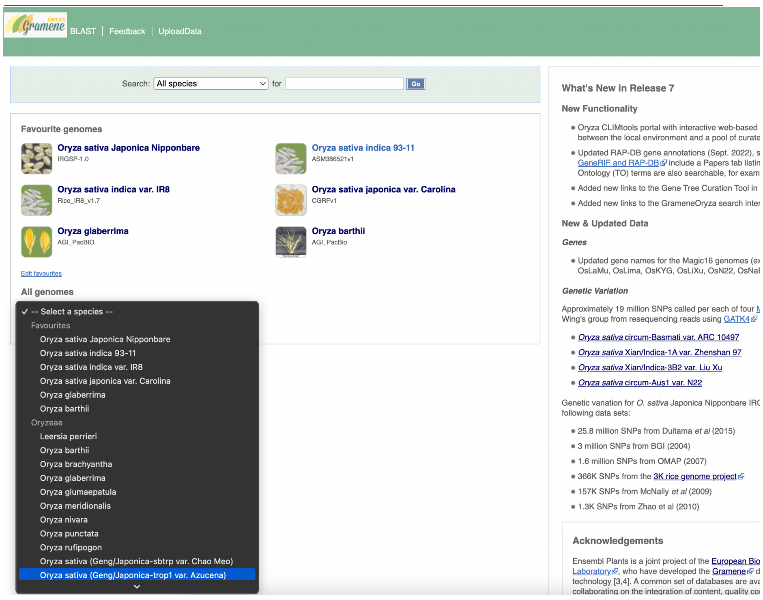

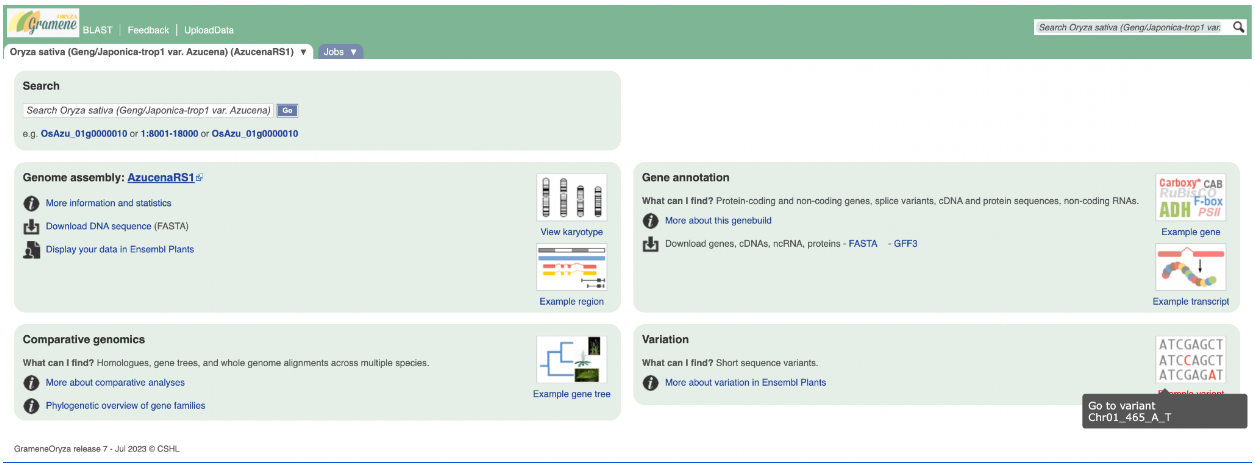

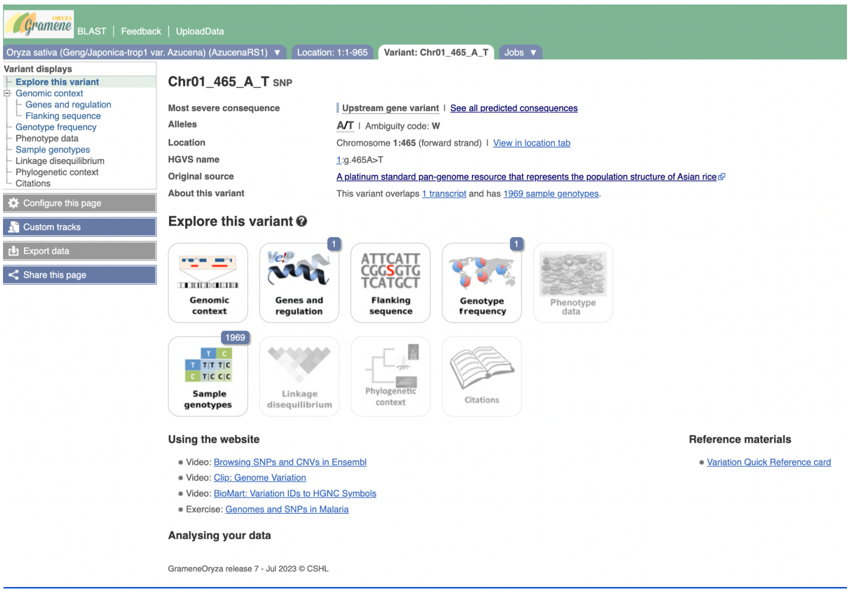

2.2. Access variation data from gene, using a MingHui63 gene name '**OsMH63_11G007180'**, as an example

Search the gene ''**OsMH63_11G007180**'' from GrameneOryza Genome Browser homepage (https://oryza-ensembl.gramene.org/index.html) search box on the top →
In result page, click gene name to get to gene page → to the left side NavBar of the genePage, click 'Variant Image' or 'Variant Table' to access variation data

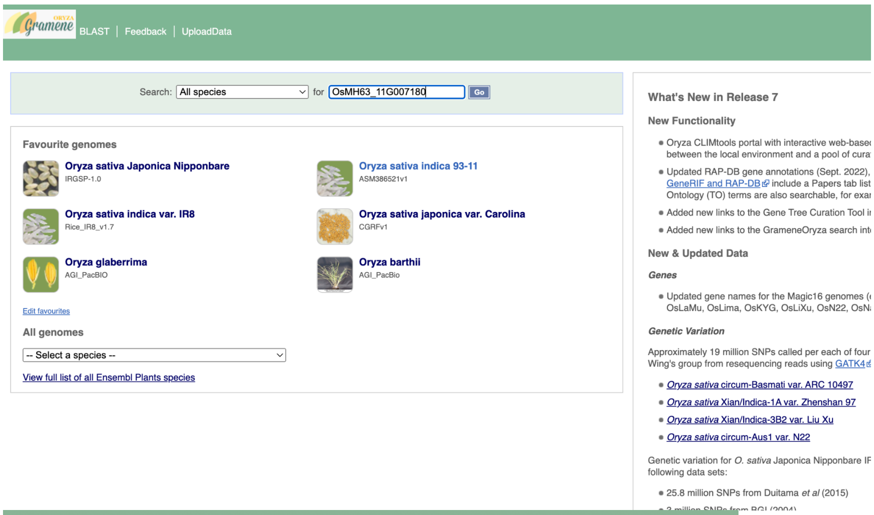

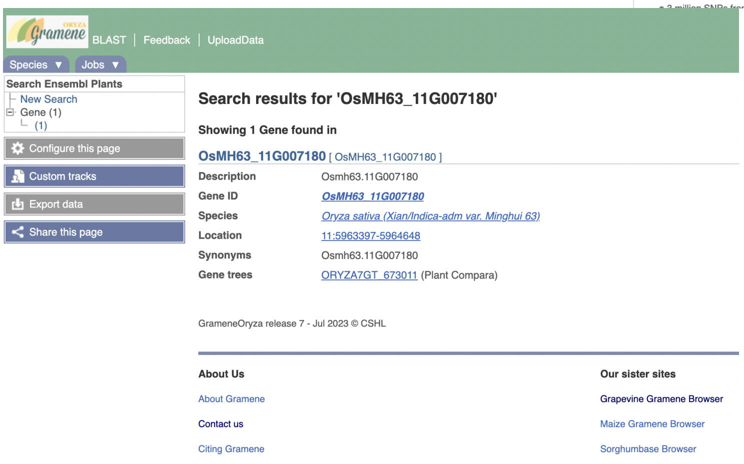

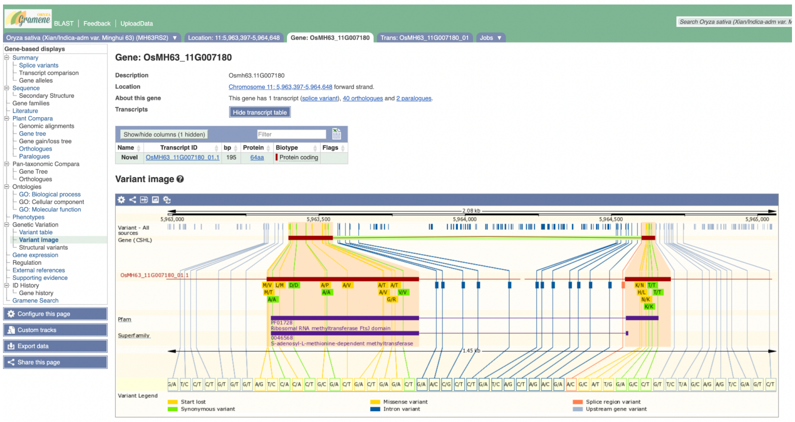

2.3. Access variation data from a region using Aus N22 genome as an example

GrameneOryza Genome Browser homepage (https://oryza-ensembl.gramene.org/index.html) select '**Oryza sativa (circum-Aus1 var. N22)**' → land on N22 homepage → enter the region of interest for example '2:80010-118000' in the search box → in the location view → click the wheel shaped icon to configure the view → after selecting the variation data in the configuration popup menu, a new track with variation will be on the location page. Each hash is a variation, different colors represent different effects → click on any hash, a popup window will give information about this SNP → select 'more about …' will take you to the variation page about this SNP.

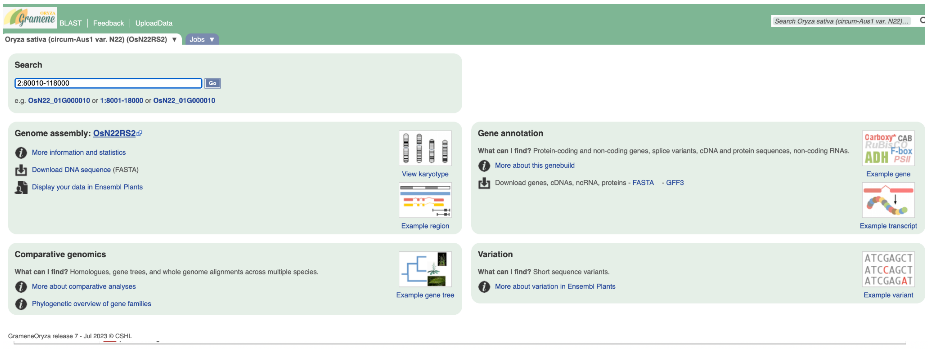
Gramene
BLAST | Feedback | UploadData
Oryza sativa (circum-Aus1 var. N22) (OsN22RS2)
Jobs
Search
2:80010-118000
Go
e.g. OsN22_01G000010 or 1:8001-18000 or OsN22_01G000010
Genome assembly: OsN22RS2
More information and statistics
Download DNA sequence (FASTA)
Display your data in Ensembl Plants
View karyotype
Example region
Gene annotation
What can I find? Protein-coding and non-coding genes, splice variants, cDNA and protein sequences, non-coding RNAs.
More about this genebuild
Download genes, cDNAs, ncRNA, proteins - FASTA - GFF3
Example gene
Example transcript
Comparative genomics
What can I find? Homologues, gene trees, and whole genome alignments across multiple species.
More about comparative analyses
Phylogenetic overview of gene families
Example gene tree
Variation
What can I find? Short sequence variants.
More about variation in Ensembl Plants
Example variant
GrameneOryza release 7 - Jul 2023 © CSHL

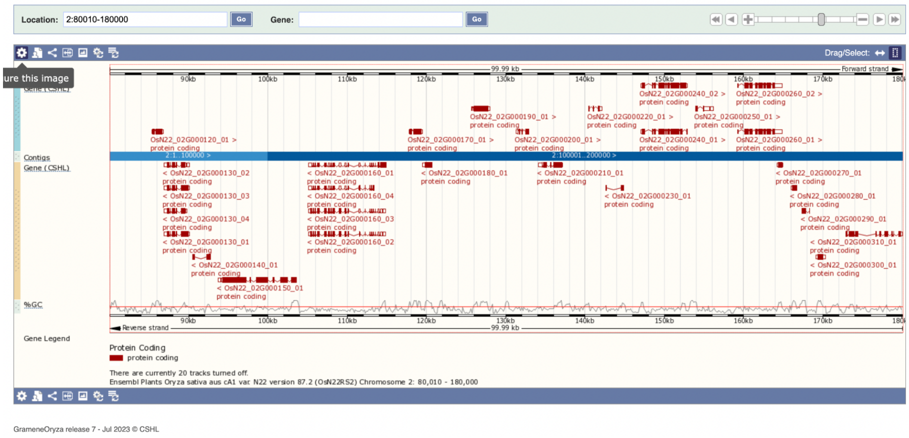
Location: 2:80010-180000
Go
Gene:
Go
Drag/Select:
Forward strand
99.99 kb
Contigs
Gene (CSHL)
%GC
Reverse strand
Gene Legend
Protein Coding
protein coding
There are currently 20 tracks turned off.
Ensembl Plants Oryza sativa aus cA1 var. N22 version 87.2 (OsN22RS2) Chromosome 2: 80,010 - 180,000
GrameneOryza release 7 - Jul 2023 © CSHL

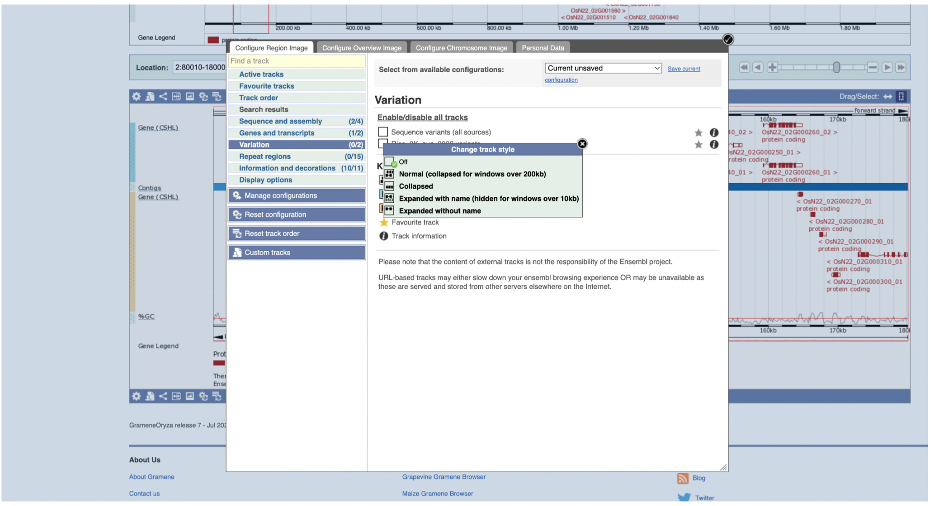
Configure Region Image
Configure Overview Image
Configure Chromosome Image
Personal Data
Find a track
Active tracks
Favourite tracks
Track order
Search results
Sequence and assembly (2/4)
Genes and transcripts (1/2)
Variation (0/2)
Repeat regions (0/15)
Information and decorations (10/11)
Display options
Manage configurations
Reset configuration
Reset track order
Custom tracks
Select from available configurations:
Current unsaved configuration
Save current configuration
Variation
Enable/disable all tracks
Sequence variants (all sources)
Change track style
Off
Normal (collapsed for windows over 200kb)
Collapsed
Expanded with name (hidden for windows over 10kb)
Expanded without name
Favourite track
Track information
Please note that the content of external tracks is not the responsibility of the Ensembl project.
URL-based tracks may either slow down your ensembl browsing experience OR may be unavailable as these are served and stored from other servers elsewhere on the Internet.
About Us
About Gramene
Contact us
Grapevine Gramene Browser
Maize Gramene Browser
Blog
Twitter

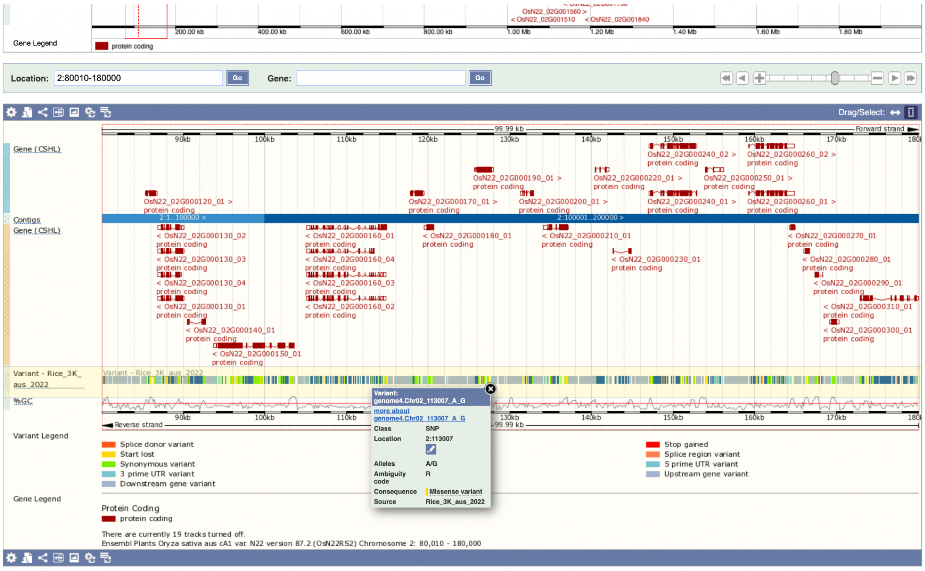

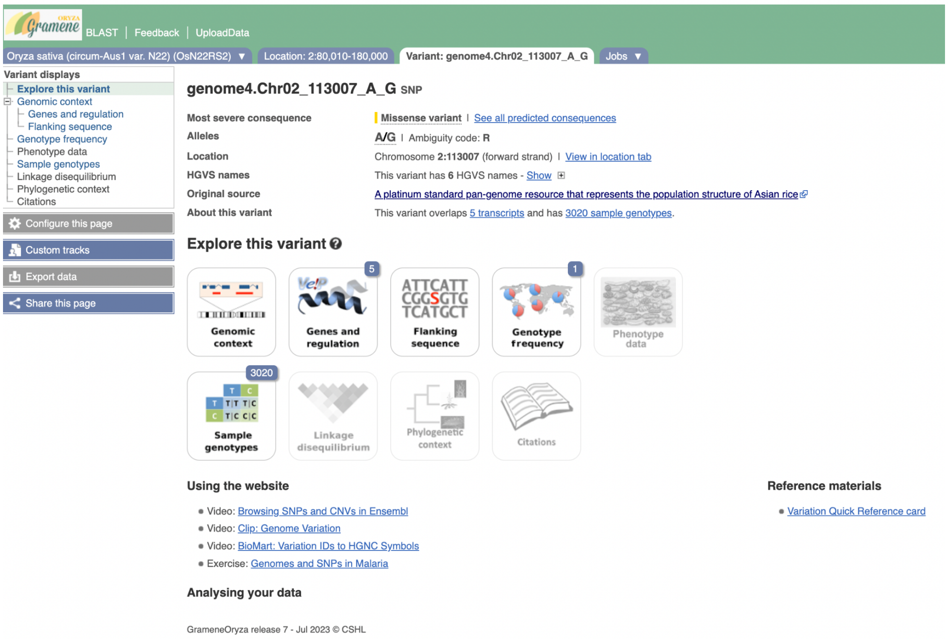

3. To know more about how to navigate GrameneOryza site and ensembl browser, the following video and tutorial maybe helpful

https://oryza.gramene.org/guides

https://www.youtube.com/@gramenedatabase3929

Ensembl Browser Workshop - Day 2 (St. Xavier's College, 29th June 2022)
